# Study on differentiating benign and malignant thyroid nodules based on CT multi-phase artificial intelligence models

**DOI:** 10.3389/fendo.2025.1738342

**Published:** 2026-01-16

**Authors:** Daoxiong Xiao, Xianzhong Wu, Peng Xie, Binglin Lai, Jianping Zhong, Junyuan Zhong, Xianjun Zeng

**Affiliations:** 1 Ganzhou Institute of Medical Imaging, Ganzhou Key Laboratory of Medical Imaging and Artificial Intelligence, Medical Imaging Center, Ganzhou People’s Hospital, The Affiliated Ganzhou Hospital, Jiangxi Medical College, Nanchang University, Ganzhou Hospital-Nanfang Hospital, Southern Medical University, Ganzhou, Jiangxi, China; 2 Department of Radiology, The First Affiliated Hospital of Nanchang University, Nanchang, Jiangxi, China

**Keywords:** artificial intelligence, computed tomography, deep learning, machine learning, radiomics, thyroid nodules

## Abstract

**Background:**

The rising global incidence of thyroid nodules necessitates improved non-invasive methods for differentiating benign from malignant lesions. However, research on artificial intelligence (AI) models using multiphase CT imaging to differentiate benign from malignant thyroid nodules is limited.

**Methods:**

This retrospective study analyzed multiphase CT data (noncontrast, arterial, and venous phases) from 604 patients with thyroid nodules confirmed by postoperative pathology. We developed and compared multiple machine learning and deep learning models using extracted radiomics features, raw 3D DICOM data, and key clinical factors (sex, age, thyroglobulin and thyrotropin levels). Model performance was evaluated using receiver operating characteristic (ROC) analysis, and Gradient-weighted Class Activation Mapping (Grad-CAM) was used for visualization.

**Results:**

Models incorporating imaging data significantly outperformed a clinical-only model (AUC = 0.811). Nomograms combining either a radiomics score (Rad-Score) or a deep learning score (AI-Score) with clinical data demonstrated the highest diagnostic accuracy. The nomogram based on Rad-Score and clinical data achieved a peak AUC of 0.885. Similarly, the AI-Score-based nomogram reached an AUC of 0.881. Both integrated approaches proved superior to models relying on a single data type.

**Conclusions:**

AI models integrating multiphase CT radiomics or deep learning features with clinical data provide a robust and highly accurate approach for differentiating benign from malignant thyroid nodules. These integrated models show significant potential for improving clinical decision-making.

## 1 Introduction

Recent studies have highlighted a rising global incidence of thyroid nodules, with an estimated adult prevalence of 24.83% and malignancy rates ranging from 7% to 15% (1, 2). In China, from 2000 to 2018, the age-standardized incidence rate (ASIR) of all cancers combined increased by 1.4% annually, with thyroid cancer showing a particularly sharp rise. By 2022, thyroid cancer had become the third most common newly diagnosed malignancy among women (3). While most nodules are benign, accurate differentiation between malignant and benign lesions remains a critical clinical challenge that directly influences management decisions, particularly decisions regarding surgery (4, 5).

Ultrasound (US) is routinely employed for thyroid nodule screening owing to its noninvasive nature, high soft-tissue contrast, and real-time imaging capabilities (2, 6, 7). Nevertheless, its accuracy can be compromised by operator dependence, artifacts from anatomical structures (such as tracheal gas or sternal interference) (8), and variations in image quality related to patient body habitus, which may diminish its ability to detect central lymph node metastases and necessitate adjunct imaging techniques (9). Similarly, although fine-needle aspiration (FNA) biopsy is fundamental to thyroid nodule evaluation (10), its accuracy is limited by potential sampling errors, including missed papillary microcarcinomas or unsampled nodules in multinodular goiter, as well as interpretative challenges due to overlapping cytomorphologic features of benign conditions such as adenomatous hyperplasia or thyroiditis that can mimic malignancy (10, 11). In addition, FNA cannot assess lymph node involvement or tumor invasiveness and often requires intraoperative frozen section analysis for definitive surgical planning (11).

In this context, computed tomography (CT) offers complementary advantages that address specific clinical gaps where US is suboptimal. CT offers several distinct advantages for thyroid imaging (12–14). It provides a clear depiction of the thyroid’s relationship with adjacent structures, especially for lesions larger than 1 cm, and improves the sensitivity for detecting central lymph node metastases by approximately 15–20% (15, 16). CT also accurately characterizes coarse and ring-shaped calcifications while reducing variability related to operator experience (17). Moreover, three-dimensional CT reconstruction enables multiplanar analysis, thereby offering a more comprehensive evaluation of complex anatomical regions. The integration of deep-learning-based radiomics further introduces a promising approach to enhance diagnostic accuracy (18).

Recent machine learning techniques have been successfully applied to ultrasound image classification of single thyroid nodule, showing promising results in automated nodule characterization (19). Most existing studies rely solely on single-plane images;however, a multi-view deep learning model recently achieved an area under the receiver operating characteristic curve (AUROC) of 0.84 (95% CI: 0.75–0.91) on an independent test set, with sensitivity (84%) and specificity (63%) surpassing those of radiologists applying the American College of Radiology Thyroid Imaging Reporting and Data System (ACR-TIRADS) to the same cohort (20). In a multicenter study, a 3D ultrasound–based deep learning system was developed to automatically segment nodules from dynamic video sequences and generate volumetric reconstructions. When used as a decision-support tool, this system improved overall diagnostic performance among radiologists, increasing the AUROC from 0.66 to 0.79; notably, even experienced readers saw their AUROC rise from 0.73 to 0.82 (21). More recently, a comparative study showed that a whole-thyroid CT radiomics model performed comparably to a lesion-based approach in differentiating benign from malignant nodules (AUROC: 0.81 vs. 0.84), suggesting that gland-level analysis may support more scalable and automated diagnostic strategies (22).

Clinically, thyroid disorders often present as multiple nodules characterized by irregular morphologies and poorly defined margins (7), complicating the accurate delineation of the full extent of the disease. Focusing on a single nodule may not capture the diffuse pathological processes observed in some thyroid malignancies. This is particularly relevant in cases involving the entire gland or a single lobe, or when no distinct tumor nodules are present, making lesion boundary delineation challenging (23). Consequently, whole-thyroid analysis allows for more robust feature extraction from both lesions and the surrounding tissue. In this study, we developed a scalable, operator-independent decision-support tool by integrating multiparametric data, including clinical variables, radiomics features, and deep learning representations—derived from whole-thyroid CT volumes, specifically designed to address clinical scenarios inadequately served by current ultrasound-centric approaches.

## 2 Methods

### 2.1 Study design and population

**Figure 1:**
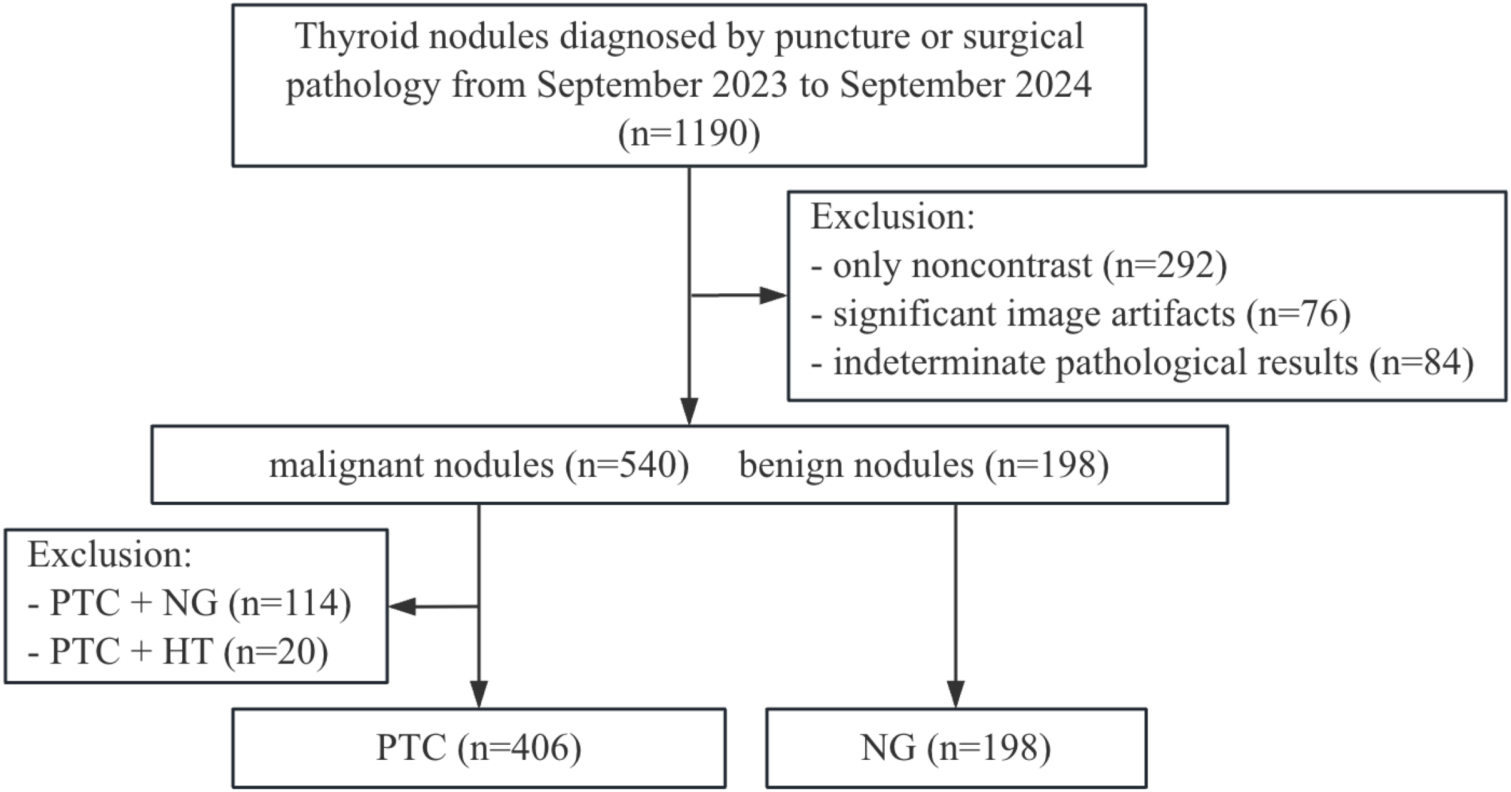
Flowchart of patient selection in this study. PTC, papillary thyroid carcinoma; NG, nodular goiter; HT, Hashimoto’s thyroiditis.

We retrospectively reviewed data from September 2023 to September 2024 in the Picture Archiving and Communication System (PACS) of the Ganzhou People’s Hospital. A total of 1,190 patients with thyroid nodules confirmed by postoperative pathology were identified. Firstly, we excluded 452 cases, including 292 cases with only noncontrast CT scans, 76 cases with significant image artifacts and 84 cases with indeterminate pathological results. This yielded a cohort of 738 cases, comprising 540 malignant and 198 benign nodules. Next, to ensure diagnostic homogeneity within each study group and because papillary thyroid carcinoma (PTC) and nodular goiter (NG) are the most common thyroid lesions encountered in surgical practice, we excluded patients exhibiting more than one distinct histopathological diagnosis in the resected thyroid tissue. This exclusion comprised 114 cases in which papillary thyroid carcinoma (PTC) coexisted with nodular goiter (NG), and 20 cases where PTC was associated with Hashimoto’s thyroiditis (HT). The final dataset included 604 cases: 406(67.22%) of papillary thyroid carcinoma and 198 (32.78%) of nodular goiter. The patient recruitment pathway for this study is illustrated in **Figure 1**. Among them, 125 cases of papillary thyroid carcinoma and 66 cases of nodular goiter had multiple nodules, accounting for 30.79% (125/406) and 33.33% (66/198) of cases, respectively. Meanwhile, we retrieved the thyroglobulin (TG) and thyroid-stimulating hormone (TSH) levels of each patient in the dataset in the Hospital Information System (HIS).

### 2.2 Image acquisition and preprocessing

All patients included in this study underwent preoperative three-phase CT, comprising noncontrast, arterial, and venous phases imaging. Scans were performed using a GE Revolution CT scanner (GE Healthcare, Milwaukee, WI, USA) with the following imaging parameters: slice thickness, 0.625 mm; pitch, 0.992:1; tube voltage, 120 kV; and automatic tube current modulation (smart mA, 200–500 mA). Intravenous contrast was administered using a power injector, delivering 60 mL of nonionic iodinated contrast material at a rate of 3.5 mL/sec. Arterial and venous phases acquisitions were initiated at 20–25 s and 55–60 s, respectively, after contrast injection. According to expert consensus on head and neck CT examination and radiation dose management issued by the Head and Neck Group of the Chinese Society of Radiology, Chinese Medical Association, the diagnostic reference level for thyroid CT (CTDIvol) is 25 mGy. The CTDIvol values for all patients in this study remained within the recommended range. During the CT scan of the neck, owing to the significant density difference between the clavicle and the surrounding soft tissues, the partial volume effect can cause band-like clavicle artifacts. To effectively reduce the interference of artifacts in thyroid imaging, it is necessary to carefully observe the lateral cervical positioning film during the scan to ensure that the mandibular pharyngeal angle is not less than 80° and that the number of vertebrae not covered by the shoulder is not less than 5.75.

All three-phase CT images were retrieved in Digital Imaging and Communications in Medicine (DICOM) format from the PACS and anonymized by removing patient identifiers, including name, sex, age, and other personal information. The anonymized DICOM images from each sequence were subsequently converted into a single Neuroimaging Informatics Technology Initiative (NIfTI) format file. To reduce the influence of factors such as equipment, scanning conditions on medical images and minimize the differences in image intensity and pixel values, we performed intensity normalization on all images and resampled them to a uniform spatial resolution of 1.0 mm × 1.0 mm × 1.0 mm. These preprocessing measures can enhance the consistency of images, thereby improving the accuracy and repeatability of subsequent analyses. A trained whole-thyroid segmentation model [based on nnU-Net (24)] was applied to the CT images for automated segmentation, generating the corresponding mask (label) images. Because the three-phase scans were completed within the same time period, the anatomical position of the thyroid gland was fixed. Due to high contrast between the thyroid and surrounding tissues on CT arterial phase images, we trained the segmentation model based on the arterial phase images and used the same mask (label) to segment the noncontrast, arterial, and venous phases images, respectively. Based on these segmentations, radiomics features and three-dimensional whole-thyroid data were extracted for further analysis.

### 2.3 Models’ construction, training and validation

In this study, the sex distribution of benign and malignant thyroid nodules was analyzed using the chi-square test. The independent-samples t-test was used to evaluate differences between benign and malignant nodules in age, thyroglobulin, and thyrotropin levels. Based on these results, a logistic regression model was constructed using the statistically significant clinical variables (age and thyroglobulin) to analyze clinical data.

**Figure 2:**
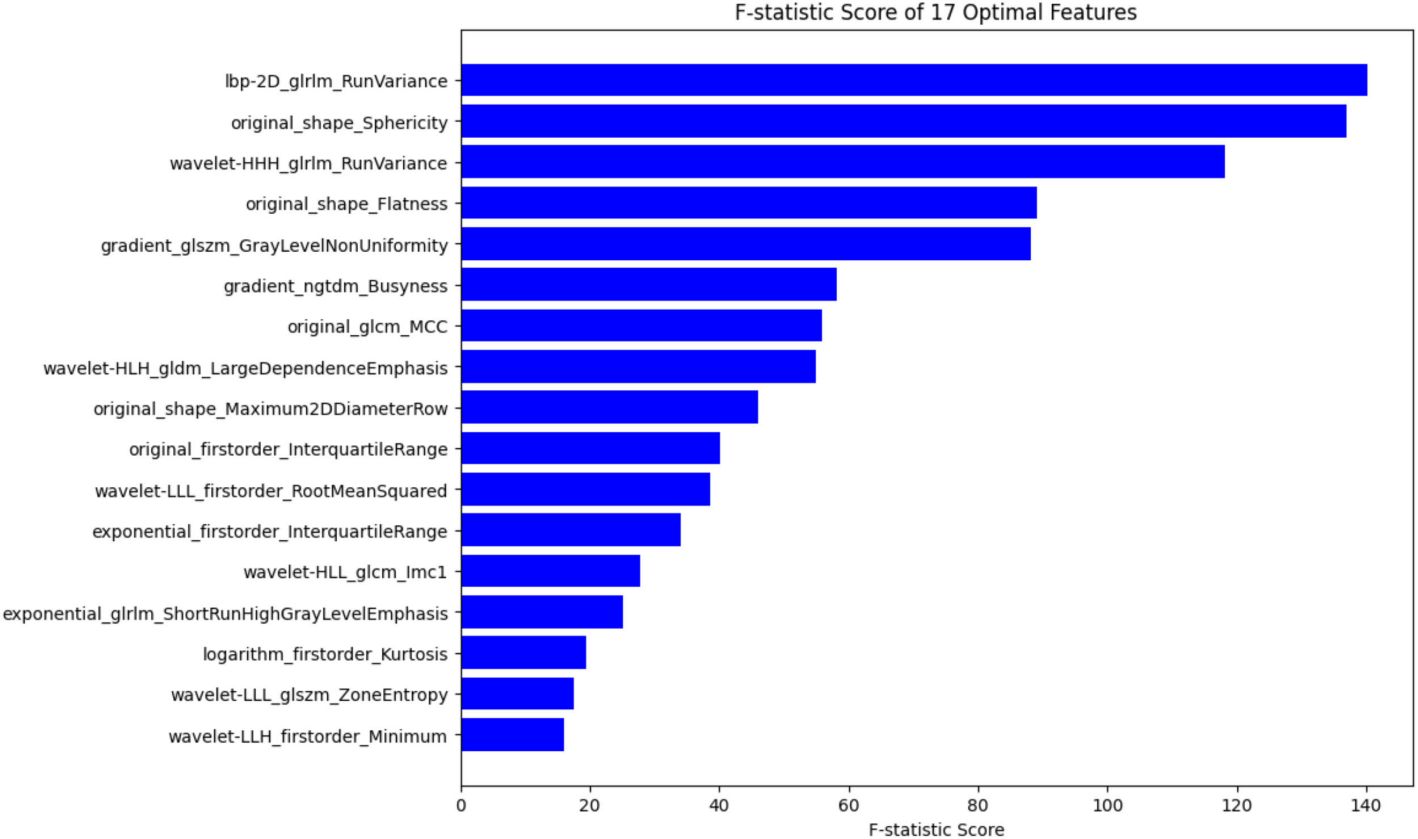
The selected features and their F-statistic scores.

Radiomics features were extracted from noncontrast, arterial, and venous phases CT images using the PyRadiomics 3.0.1 open-source Python package (https://github.com/AIM-Harvard/pyradiomics#readme). Radiomics features were extracted from both the original CT images and a range of filtered images—including wavelet, Laplacian of Gaussian (LoG), square, square root, logarithm, exponential, and gradient transformations—as enabled by the enableAllImageTypes() and enableAllFeatures() functions in PyRadiomics. This yielded an initial feature set of 1,414 features per imaging phase. Feature selection was conducted in a two-step process. First, Least Absolute Shrinkage and Selection Operator (LASSO) regression was applied to reduce dimensionality and identify a subset of potentially relevant features. Subsequently, the K-best feature selection method (based on univariate F-statistic analysis) was employed, and 5-fold cross-validation was applied to determine the optimal number of features (K = 17) from the LASSO-refined feature pool. The selected features and their F-statistic scores are shown in **Figure 2**. Based on the selected features, several machine learning models—including k-nearest neighbors (KNN), logistic regression (LR), decision tree (DT), random forest (RF), support vector machine (SVM), multilayer perceptron (MLP), and Gaussian naive Bayes (GNB)—were trained and evaluated. The optimal model was identified based on Delong test results. Logistic regression analysis was applied to the selected radiomics features to calculate the radiomics-based risk score (Rad-Score).

Multiple deep learning architectures, including ResNet, Contrastive Language-Image Pre-training (CLIP), Siamese network, 3D U-Net, and Transformer, were developed and trained on noncontrast, arterial, and venous phases images. After systematic performance evaluation, the 3D U-Net model demonstrated superior capability in capturing the three-dimensional features of thyroid nodules and was selected as the final deep learning model. The chosen model architecture (implemented using the MONAI framework, https://project-monai.github.io/) was configured with an input size of 128×128×64, batch size of 4, Adam optimizer (learning rate = 1e-4), and ReduceLROnPlateau scheduler (mode = ‘min’, patience = 10, factor = 0.5). The model was trained for 1000 epochs with comprehensive data augmentation techniques including rotation, flipping, zooming, affine transformations, and noise injection. Weight initialization utilized partial transfer learning from a ResNet-18 backbone pretrained on natural image datasets. The trained model generated probability estimates for malignancy that were recorded as the AI-derived risk score (AI-Score).

Based on the clinical variables (age and thyroglobulin level), RScore, and AIScore, combined predictive models were constructed, including two combined approaches: (a) clinical data plus Rad-Score and (b) clinical data plus AI-Score. Multiple machine-learning classifiers (KNN, LR, DT, RF, SVM, MLP, and GNB) were successively trained on each CT phase to develop these combined models. Additionally, a nomogram model was developed to quantitatively evaluate and visually represent the diagnostic performance of the integrated approaches across all imaging phases to provide a comprehensive and quantitative tool for differentiating benign from malignant thyroid nodules. Gradient-weighted Class Activation Mapping (Grad-CAM) was used to visualize the key regions involved in model decision-making, enhance interpretability, and aid observers in understanding the model’s recognition process.

### 2.4 Statistical processing

The Shapiro-Wilk test was used to assess the normality of the data distribution. Continuous variables following a normal distribution are expressed as mean ± standard deviation (Mean ± SD) and compared using the independent-samples t-test. Non-normally distributed continuous variables are reported as median (interquartile range) [Median (IQR)] and compared using the Mann-Whitney U test. Clinical data were analyzed using the chi-square test or t-test. Receiver operating characteristic (ROC) curve analysis was performed to assess the predictive model for differentiating benign from malignant thyroid nodules in both the training and testing cohorts. The performance metrics included sensitivity, specificity, accuracy, positive predictive value (PPV), negative predictive value (NPV), and area under the ROC curve (AUC). The AUC values were compared using the DeLong test. Statistical significance was defined as a two-sided p-value < 0.05. All statistical analyses were conducted using Python (version 3.11.7) with SciPy (version 1.11.4) and scikit-learn (version 1.5.2) or R (version 4.4.1).

### 2.5 Ethical approval

This study was reviewed and approved by the Ethics Committee of Ganzhou People’s Hospital (Approval No. PJB2025-302-01). The requirement for informed consent was waived due to the retrospective study design. All procedures were performed in accordance with the Declaration of Helsinki and relevant national regulations.

## 3 Results

### 3.1 Patients and clinical characteristics

This study included 604 patients, including 406 with papillary carcinoma and 198 with nodular goiter. Among these, 125 (30.79%) patients had papillary carcinoma, and 66 (33.33%) had nodular goiter presenting with multiple nodules. In terms of sex distribution, the papillary carcinoma group included 309 women and 97 men, while the nodular goiter group comprised 155 women and 43 men. No significant differences in sex distribution were observed between the groups. The age of patients with papillary carcinoma ranged from 13 to 82 years (41.14 ± 11.44 years), whereas the age of patients with nodular goiter ranged from 13 to 73 years (51.03 ± 11.23 years). The differences in age between the groups were statistically significant. Thyroglobulin levels ranged from 0.04 to 500 ng/mL (14.49[6.09-27.00] ng/mL) in the papillary carcinoma group and from 0.338 to 500 ng/mL (57.95[19.68-157.93] ng/mL) in the nodular goiter group, with a statistically significant difference between the two groups. Thyroid-stimulating hormone levels ranged from 0.005 to 19.76 μIU/mL (1.67[1.05-2.39]μIU/mL) in the papillary carcinoma group and from 0.01 to 188.16 μIU/mL (1.38[0.81-2.13]μIU/mL) in the nodular goiter group, with no significant difference between groups. The clinical characteristics of patients in the nodular goiter and papillary carcinoma cohorts were summarized in **Table 1**.

**Table 1:** Characteristics of patients in NG and PTC cohorts.

Characteristic	NG (n=198)	PTC (n=406)	p
Age (years)			<0.001
Mean ± SD	51.03 ± 11.23	41.14 ± 11.44	
Sex, No. (%)			0.623
Male	43 (21.72)	97 (23.89)	
Female	155 (78.28)	309 (76.11)	
Number of nodules, No. (%)			0.590
Single	132 (66.67)	281 (69.21)	
Multiple	66 (33.33)	125 (30.79)	
TG levels (ng/mL)			<0.001
Median (IQR)	57.95[19.68-157.93]	14.49[6.09-27.00]	
TSH levels (μIU/mL)			0.266
Median (IQR)	1.38[0.81-2.13]	1.67[1.05-2.39]	

Data are number of patients and percentage if not specified. Significant differences are highlighted in boldface. NG, nodular goiter; PTC, papillary thyroid cancer; TG, Thyroglobulin; TSH, Thyroid-stimulating hormone.

### 3.2 Model evaluation and visualization

**Figure 3:**
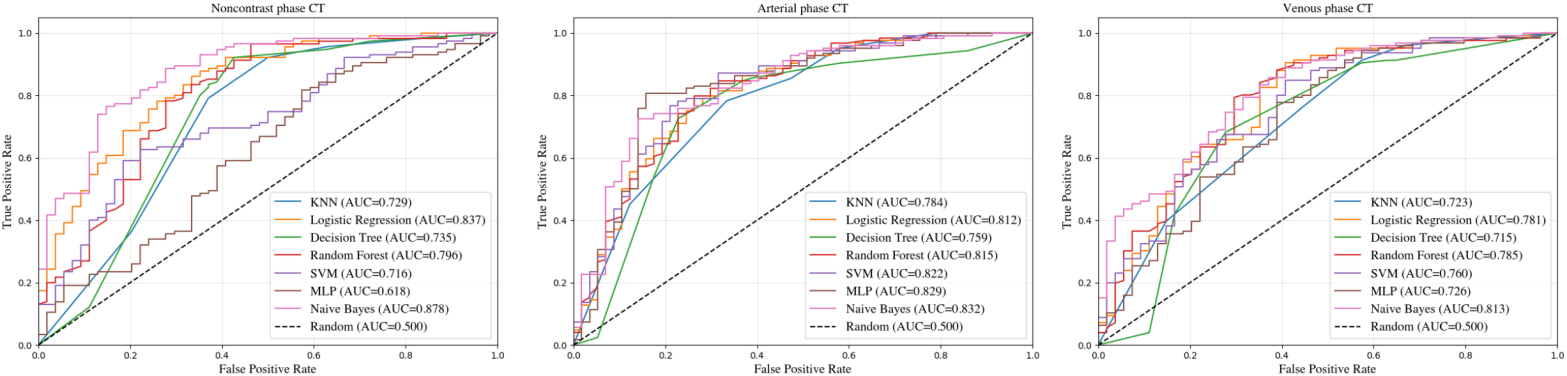
ROC curves based on radiomics.

A total of 1,414 radiomics features were extracted from thyroid CT images acquired during the noncontrast, arterial, and venous phases. These features were reduced to 17 dimensions for the subsequent analysis. Classification models, including KNN, LR, DT, RF, SVM, MLP, and GNB, were evaluated using the ROC curve analysis (**Figure 3**). Among these, GNB demonstrated the highest performance, with AUC values of 0.878, 0.832, and 0.813 for the noncontrast, arterial, and venous phases, respectively.

**Figure 4:**
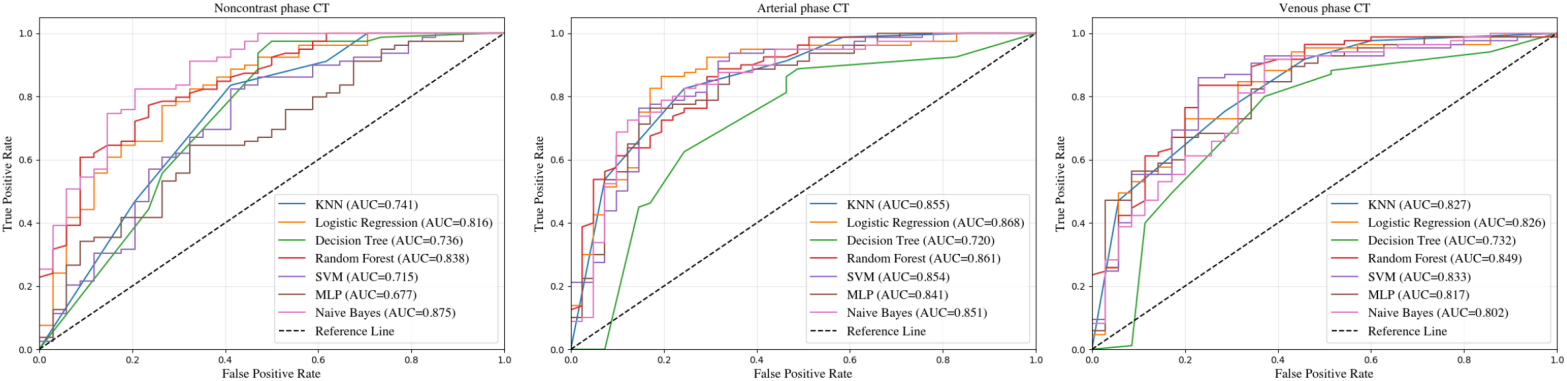
ROC curves based on radiomics integrated with clinical variables (sex, age, and thyroglobulin levels).

The integration of radiomics scores (Rad-Score) with clinical variables (sex, age, and thyroglobulin levels) improved the classification performance. ROC analysis (**Figure 4**) indicated that GNB, LR, and RF exhibited superior and more stable performances across all three phases. Specifically, GNB achieved an AUC of 0.875 in the noncontrast phase, LR reached the highest AUC of 0.868 in the arterial phase, and RF obtained an AUC of 0.849 in the venous phase. In contrast, the KNN and DT models demonstrated lower and less consistent AUC values, respectively.

A nomogram integrating radiomics features (Rad-Score) with clinical variables demonstrated robust diagnostic performance across all contrast-enhanced CT phases. For noncontrast CT, the model achieved a concordance index (C-index) of 0.874 and an area under the receiver operating characteristic curve (AUC) of 0.876, with a sensitivity of 0.926, specificity of 0.619, positive predictive value (PPV) of 0.836, and negative predictive value (NPV) of 0.8. On arterial phase CT, performance slightly improved (C-index, 0.883; AUC, 0.885), with a sensitivity of 0.933, specificity of 0.631, PPV of 0.838, and NPV of 0.822. Similar results were observed on venous phase CT (C-index, 0.878; AUC, 0.879), with a sensitivity of 0.931, specificity of 0.619, PPV of 0.835, and NPV of 0.811. The performance metrics of each CT phase were summarized in **Table 2**.

**Table 2:** Performance metrics comparison of nomogram incorporating radiomics (Rad-Score) and clinical features.

Metric	Noncontrast CT	Arterial CT	Venous CT
C-index	0.874	0.883	0.878
AUC	0.876	0.885	0.879
Sensitivity	0.926	0.933	0.931
Specificity	0.619	0.631	0.619
PPV	0.836	0.838	0.835
NPV	0.800	0.822	0.811

PPV, Positive Predictive Value; NPV, Negative Predictive Value.

**Figure 5:**
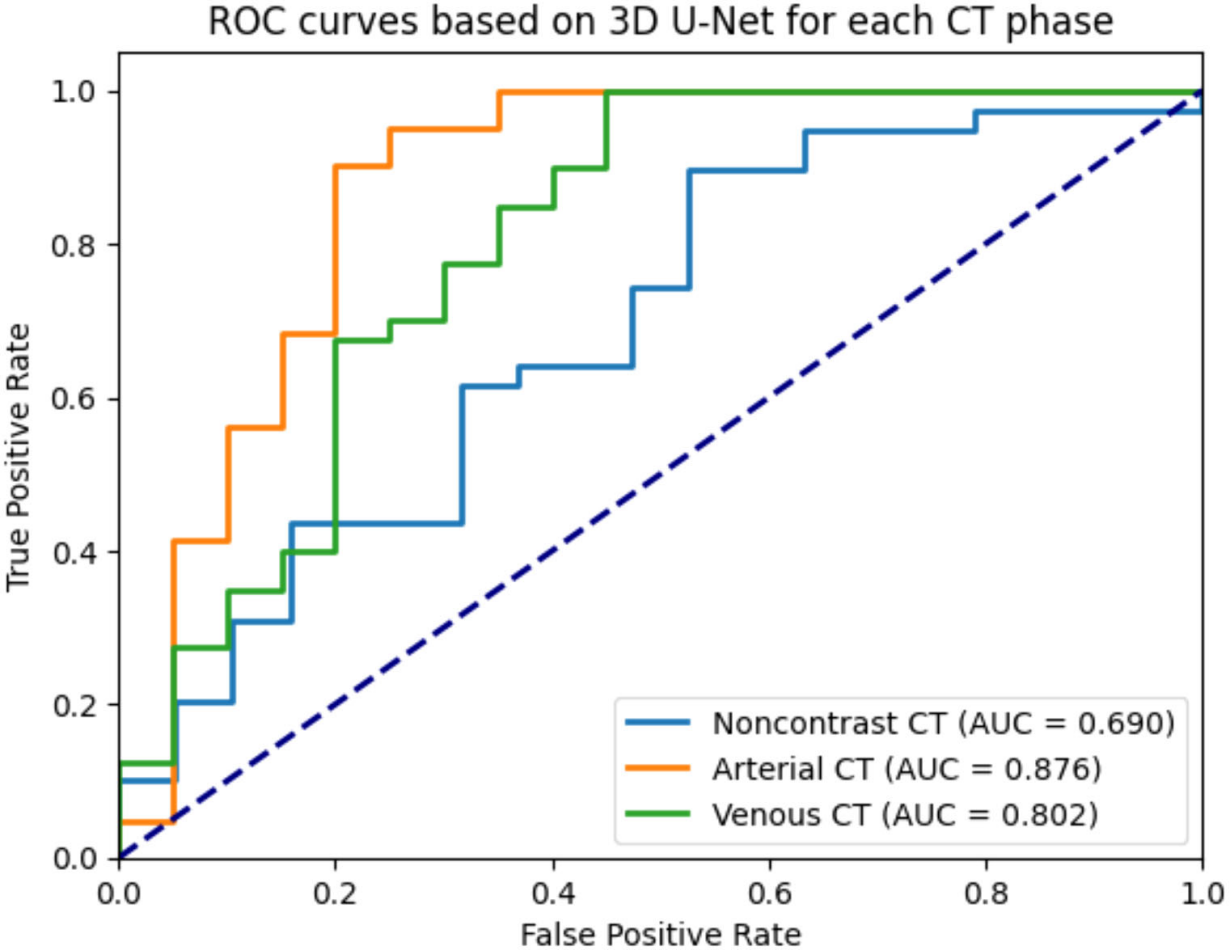
ROC curves based on 3D U-Net for each CT phase.

The 3D U-Net deep learning model achieved AUC values of 0.690, 0.876, and 0.802 in the noncontrast, arterial, and venous phases, respectively (shown in **Figure 5**). The ROC curve was relatively flat in the noncontrast phase, indicating a limited diagnostic efficacy. In contrast, the arterial phase exhibited a steep ROC curve, suggesting high true-positive rates and low false-positive rates. The venous phase demonstrated a rapid increase in the ROC curve at low false-positive rates, reflecting acceptable overall diagnostic capability.

**Figure 6:**
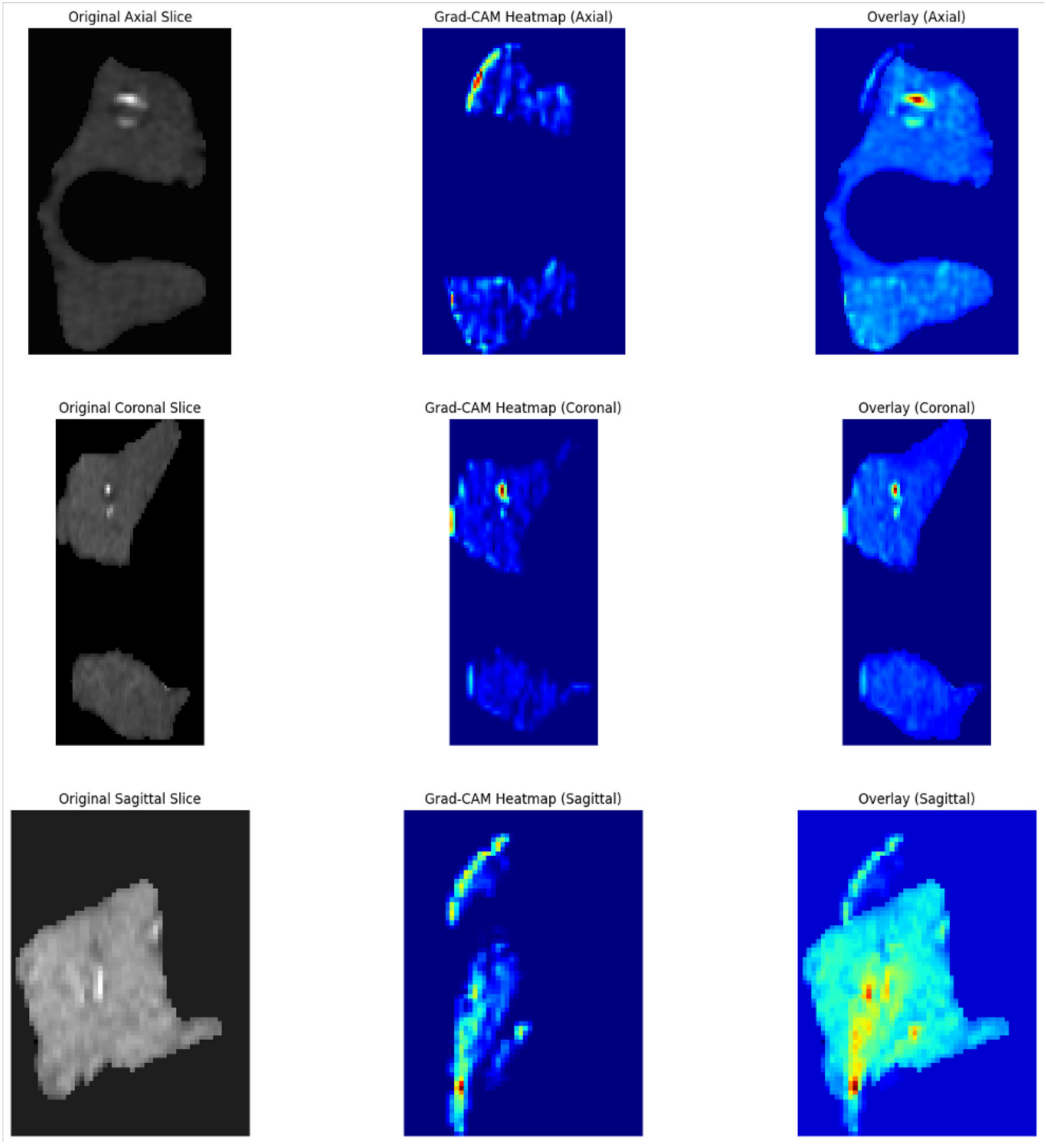
Grad-CAM visualization generated by 3D U-Net on noncontrast phase.

Gradient-weighted Class Activation Mapping (Grad-CAM) visualization from the optimal model (3D U-Net) highlighted the spatial regions most influential in classification decisions, with color intensity indicating the degree of model attention. As shown in **Figure 6**, the model’s attention patterns closely align with those of human observers.

A nomogram incorporating deep learning–based AI scores (AIScore) and clinical features demonstrated robust diagnostic performance across all CT phases. For noncontrast CT, the model achieved a concordance index (C-index) of 0.875 and an area under the curve (AUC) of 0.878, with a sensitivity of 0.910, specificity of 0.656, positive predictive value (PPV) of 0.847, and negative predictive value (NPV) of 0.777. For arterial phase CT, the C-index was 0.871 and AUC was 0.873, with a sensitivity of 0.933, specificity of 0.611, PPV of 0.830, and NPV of 0.818. For venous phase CT, the model yielded a C-index of 0.879 and AUC of 0.881, with a sensitivity of 0.921, specificity of 0.649, PPV of 0.845, and NPV of 0.797. A summary of performance metrics across all CT phases is provided in **Table 3**.

**Table 3:** Performance metrics comparison of nomogram incorporating AIScore and clinical features.

Metric	Noncontrast CT	Arterial CT	Venous CT
C-index	0.875	0.871	0.879
AUC	0.878	0.873	0.881
Sensitivity	0.91	0.933	0.921
Specificity	0.656	0.611	0.649
PPV	0.847	0.83	0.845
NPV	0.777	0.818	0.797

## 4 Discussion

In this study, we developed and evaluated a multimodal CT-based classification framework integrating clinical variables, handcrafted radiomics (Rad-Scores), and deep learning–derived features (AI-Scores) from whole-thyroid multiphase CT scans. the models demonstrated strong discriminatory performance for differentiating benign from malignant thyroid nodules. The findings support CT-based AI as a promising complementary tool in thyroid nodule assessment.

Although US remains the first-line imaging modality for thyroid nodules evaluation due to its accessibility, lack of ionizing radiation, and high spatial resolution for superficial structures, it is inherently limited by operator dependency, inter-scanner variability, and challenges in characterizing complex or deeply located nodules (4, 25). Recent advances have demonstrated that AI-enhanced US can achieve notable diagnostic performance: for instance, a multi-view deep learning model reported an AUROC of 0.84 with sensitivity of 84% and specificity of 63%, outperforming radiologists applying ACR-TIRADS on the same cohort (20). Moreover, a 3D US-based deep learning system improved radiologists’ diagnostic AUROC from 0.66 to 0.79 and even experienced readers saw gains from 0.73 to 0.82 by enabling volumetric nodule reconstruction from dynamic video sequences (21). Despite these advances, US-based AI systems remain constrained by image quality heterogeneity across institutions and the reliance on single-plane or limited-volume inputs, which may hinder robustness in real-world settings.

In contrast, CT offers several distinct advantages that are particularly relevant in specific clinical scenarios. First, CT provides superior visualization of coarse calcifications, extrathyroidal extension, and retrosternal or substernal nodules—features often poorly assessed by US but critical for malignancy risk stratification (25). Second, CT imaging is less susceptible to operator-induced variability, enabling more reproducible radiomic feature extraction—a key prerequisite for scalable AI deployment. Third, the whole-thyroid coverage afforded by CT facilitates gland-level analysis, which is especially valuable in multifocal disease or diffuse infiltrative carcinomas where focal US assessment may miss subtle lesions. Indeed, A recent comparative study demonstrated that a whole-thyroid CT radiomics model achieved an AUC of 0.81 in differentiating benign from malignant thyroid nodules—comparable to the lesion-based model (AUC: 0.84). No significant difference in AUC was observed between the two region-of-interest strategies, indicating that whole-thyroid analysis provides equivalent diagnostic performance by integrating comprehensive information from both the nodule and surrounding parenchyma while reducing manual segmentation errors. This approach supports fully automated thyroid segmentation and enhances the feasibility of clinical software applications (22).

Our CT-based framework builds upon these strengths. Among seven classifiers, Gaussian Naive Bayes (GNB) achieved peak performance in the noncontrast phase (high AUC and sensitivity), while the 3D U-Net model performed best in the arterial phase (AUC = 0.876), likely due to improved lesion-to-background contrast during vascular enhancement. The phenomenon consistently reported in multiphase CT imaging of thyroid malignancies, where hypervascularity is a known hallmark of papillary thyroid carcinoma (12, 26, 27). Grad-CAM visualizations confirmed that model predictions were anchored in biologically plausible regions of nodule heterogeneity, such as irregular margins and internal calcifications, features long recognized in radiological literatures as suspicious for malignancy (28, 29). Our CT-based artificial intelligence model demonstrated an AUC of up to 0.885, comparable to or slightly exceeding that reported for leading ultrasound-based AI systems (30–32). Importantly, CT provides complementary information not readily accessible with ultrasound, including comprehensive three-dimensional anatomical context, assessment of retro tracheal or substernal extension, and more precise characterization of coarse or rim calcifications that may be underestimated or obscured on ultrasound due to acoustic shadowing or limited field of view. In light of these capabilities, CT-based AI is not intended as a first-line screening tool but rather as a complementary modality in specific clinical scenarios, such as preoperative staging, evaluation of large or substernal nodules, assessment of extrathyroidal extension, and cases with indeterminate or discordant ultrasound findings. This approach aligns with recent recommendations advocating multimodal integration to support precision management in thyroid oncology.

Although this study has many advantages, it has some limitations. First, this work represents a proof-of-concept study based on single-center data; thus, its external validity may be limited. Future multicenter studies that include mixed-pathology cases are needed to enhance generalizability and real-world applicability. Second, the retrospective study design may have resulted in incomplete data or record bias, and future prospective studies may have further validated the findings. In addition, the biological interpretation of radiomics features remains challenging, and future studies should attempt to integrate them with genomic and pathological data. Finally, despite the inclusion of multiple models, there may still be more advanced models or techniques (such as complex deep learning networks and ensemble learning methods) that perform better in distinguishing PTC from NG, which need to be explored in future studies.

## 5 Conclusion

This study provides a new method for the application of machine learning and deep learning based on global thyroid CT images for the identification of benign and malignant thyroid nodules. By integrating multiphase CT–based radiomics or deep learning features with clinical data provides a reliable and accurate method for differentiating benign from malignant thyroid nodules. The combined models outperform clinical assessment alone, offering quantitative and interpretable tools for non-invasive risk stratification. These AI-driven approaches hold strong potential to enhance diagnostic precision and support clinical decision-making in thyroid nodule management.

## Data Availability

The raw data supporting the conclusions of this article will be made available by the authors, without undue reservation.
